# Cosmic Ray Irradiation of Interstellar Ices on Sulfur-Rich Grains: A Possible Source of Sulfur-Bearing Molecules

**DOI:** 10.1021/acsearthspacechem.5c00036

**Published:** 2025-05-07

**Authors:** Duncan V. Mifsud, Zuzana Kaňuchová, Olivier Auriacombe, Péter Herczku, Danna Qasim, Sándor T. S. Kovács, Richárd Rácz, Béla Sulik, Zoltán Juhász, István Rajta, István Vajda, Sándor Biri, Robert W. McCullough, Sergio Ioppolo, Ujjwal Raut, Nigel J. Mason

**Affiliations:** 1 Centre for Astrophysics and Planetary Science, School of Physics and Astronomy, University of Kent, Canterbury CT2 7NH, United Kingdom; 2 HUN-REN Institute for Nuclear Research (Atomki), Debrecen H-4026, Hungary; 3 Astronomical Institute, Slovak Academy of Sciences, Tatranská Lomnica SK-059 60, Slovakia; 4 Department of Microtechnology and Nanoscience, Chalmers University of Technology, Göteborg 412 96, Sweden; 5 Southwest Research Institute, San Antonio, Texas 78238, United States; 6 Department of Physics and Astronomy, School of Mathematics and Physics, Queen’s University Belfast, Belfast BT7 1NN, United Kingdom; 7 Centre for Interstellar Catalysis, Department of Physics and Astronomy, Aarhus University, Aarhus DK-8000, Denmark

**Keywords:** astrochemistry, radiation chemistry, sulfur, interstellar ices, laboratory experiments, infrared spectroscopy

## Abstract

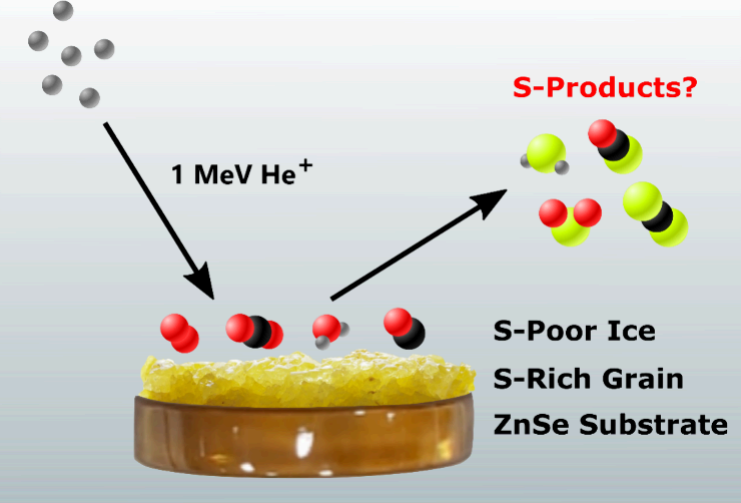

The major reservoir of sulfur in dense interstellar clouds is still largely unknown, although a growing body of evidence suggests that it may exist in a refractory form (i.e., as minerals or allotropes of the element). Therefore, it is possible that the irradiation of sulfur-free interstellar ices on top of sulfur-rich refractory grain components by cosmic rays or stellar winds may result in the formation of simple inorganic sulfur molecules that could be readily detected by ground- or space-borne telescopes. In this study, we have irradiated neat ices of O_2_, CO, CO_2_, and H_2_O on top of layers of allotropic sulfur at 20 K using 1 MeV He^+^ ions as a mimic of space radiation. Experiments with CO_2_ and H_2_O ices were also repeated at 70 K to provide data obtained under conditions more relevant to icy bodies in the outer solar system for comparative purposes. We have found qualitative mid-infrared spectroscopic evidence for the synthesis of SO_2_, CS_2_, OCS, and H_2_SO_4_ hydrates, but not H_2_S, in our experiments and have quantified the efficiency of their formation by calculating the *G*-value (i.e., the number of molecules formed per 100 eV of energy deposited) for each ice-refractory system. Overall, SO_2_ and CS_2_ are the most commonly observed products in our experiments, although the highest *G*-value was that for H_2_SO_4_ hydrates formed as a result of the irradiation of H_2_O ice on top of sulfur at 70 K. An important outcome of our study is that our experimental results are consistent with recent observational surveys that suggest SO_2_ formation in interstellar ices proceeds primarily *via* an “energetic” route involving radiolytic processes, while OCS forms as a result of “nonenergetic” processes such as atom or radical addition reactions.

## 1 Introduction

The astrochemistry of sulfur has long posed problems to the scientific community.^1^ In interstellar media, for instance, it is known that the observable abundance of sulfur in diffuse regions largely aligns with its expected cosmic abundance, but that it is depleted by about two orders of magnitude within dense molecular clouds.^2−7^ The principal drivers and mechanisms behind this so-called sulfur depletion problem are not fully understood, though a number of previous experimental and computational studies have suggested various reservoirs that may accommodate this missing sulfur. For instance, modeling efforts by Laas and Caselli^8^ suggested that organosulfur molecules embedded within interstellar icy grain mantles could account for a large percentage of sulfur. Other studies have proposed gas-phase atoms as the dominant sulfur species, which could be converted to H_2_S by surface-catalyzed reactions on dust grains or ice surfaces.^9,10^ However, the as-yet nondetection of solid H_2_S within dense molecular clouds^11,12^ seems to suggest that other potential interstellar sulfur reservoirs should be sought. More recently, sulfur-bearing salts such as NH_4_SH have been suggested to be a potential sink for interstellar sulfur.^13,14^

Ruffle et al.^6^ proposed that a potential major sink of gas-phase sulfur during the evolution of diffuse interstellar clouds into denser structures is the Coulomb-enhanced freeze-out of S^+^ ions onto negatively charged dust grains. Although the subsequent chemical reactions in which grain-adsorbed sulfur atoms are still somewhat speculative, it is conceivable that atom addition reactions may proceed to yield various allotropes. Indeed, evidence for the reaction of sulfur atoms to yield S_2_ and thence S_2*n*_ allotropes can be found in previous experimental studies.^15−18^ Comprehensive studies by Shingledecker et al.^19^ that incorporated both cosmic ray-induced chemistry and fast, nondiffusive radical combination reactions of the type previously described by Garrod et al.^20^ have also demonstrated that sulfur chemistry in interstellar icy grain mantles could produce large quantities of S_8_. These theoretical results have been corroborated by experimental studies that have shown that the processing of astrophysical ice analogs containing simple inorganic sulfur-bearing molecules by ions, electrons, or ultraviolet photons results in the formation of refractory sulfur-rich residues, which likely include large allotropes.^21−27^

Although the observation of sulfur allotropes on interstellar dust grains is very challenging due to their low absorbances across the mid-infrared range,^28^ their possible existence in such environs is perhaps reinforced by their detection in asteroid 162173 Ryugu and comet 67P/Churyumov-Gerasimenko.^29,30^ An alternative possible sink for the depletion of interstellar sulfur is its inclusion in mineral phases, primarily as metal sulfides. Such minerals are thought to be among the earliest synthesized in the history of mineralogical evolution and, indeed, so-called GEMSs (silicate glass with embedded metal and sulfides) have been detected in interstellar dust grains found preserved in chondritic meteorites.^31^ Moreover, observations of (post)asymptotic giant branch stars have revealed a high abundance of sulfide minerals, particularly those of iron and magnesium.^32,33^ Work by Kama et al.^34^ has also suggested that such minerals could account for a large majority (>80%) of the sulfur in protoplanetary disks around young stars.

Either way, the results of these recent studies suggest that a considerable fraction of the missing interstellar sulfur could be locked away as refractory solid components of dust grains (i.e., allotropes or minerals). Such an implication is important, as this sulfur may interact chemically with an overlying ice mantle to produce new molecules. This is especially true when galactic cosmic rays interact with interstellar icy grain mantles, since such an injection of energy is known to lead to the formation of a plethora of new species, some of which may be complex organic molecules that are relevant to the emergence of life.^35−37^ Previous laboratory work has demonstrated that the irradiation of interstellar ice analogs on top of carbon-rich grain analogs leads to the formation of a number of carbon-bearing inorganic molecules, most prominently CO and CO_2_. For instance, Mennella et al.^38^ demonstrated that the irradiation of H_2_O ice on top of hydrogenated carbon grains simulating interstellar dust particles using 30 keV He^+^ ions resulted in the synthesis of both CO and CO_2_. This result was echoed by Raut et al.,^39^ who observed the formation of CO_2_ after the 100 keV proton irradiation of H_2_O ice on amorphous carbon. Gomis and Strazzulla^40^ went on to demonstrate that the synthesis of CO and CO_2_ in this manner does not depend on the chemical nature of the underlying residue, so long as it is carbon-rich. Finally, the studies of Mennella et al.^41^ and Fulvio et al.^42^ showed that this chemistry could also be induced by ultraviolet photons, as well as ions.

Given that sulfur may be an important component of refractory interstellar grains, either as allotropes or minerals, and in light of the results of previous analogous studies making use of refractory carbon,^38−42^ it occurred to us that the interaction of galactic cosmic rays with icy material on top of sulfur-rich dust grains may lead to the synthesis of new molecules at the ice-refractory interface, which may be detectable by ground- or space-borne observatories and telescopes. This idea is reinforced by the recent work of Ferrari et al.,^43^ which revealed that the fragmentation of S_8_ under simulated astrophysical conditions is facile, thus further hinting at a potentially important role for allotropic sulfur in interstellar sulfur chemistry. At present, only two sulfur-bearing molecules have been either definitively or tentatively detected within interstellar icy grain mantles: OCS and SO_2_.^11,12^ Indeed, it is possible that this mechanism of refractory sulfur volatilization is an important contributor to the presence of sulfur-bearing molecules in interstellar ices, and that such molecules may represent the starting point for a relatively rich subsequent organic chemistry leading to ever more complex sulfur molecules.^44−48^

In this study, we probed the formation of sulfur-bearing molecules of interest to interstellar chemistry as a result of the cosmic ray irradiation of sulfur-free ices on top of sulfur-rich dust grains. Specifically, we have irradiated neat ices of O_2_, CO, CO_2_, and H_2_O on top of allotropic sulfur at 20 K using 1 MeV He^+^ ions as mimics of galactic cosmic rays. In the case of CO_2_ and H_2_O ices deposited on top of sulfur, experiments were also performed at 70 K to allow for a comparison of the radiation-induced sulfur chemistry occurring under interstellar conditions to that occurring on the surfaces of higher-temperature outer solar system objects. Sulfur-bearing radiolytic products have been identified through mid-infrared absorption spectroscopy, and their formation efficiencies have been quantified through the so-called *G*-value, which is a measurement of the number of molecules formed per 100 eV of energy dosed into the irradiated target. We note that several related but somewhat distinct definitions exist for the *G*-value; however, we have followed the example of a number of previous studies that define it by considering the total energy dosed into both the adsorbate and the adsorbent materials,^49−53^ which, in our case, correspond to the icy material and the underlying refractory sulfur. Finally, our results are discussed in the context of their applicability to sulfur astrochemistry and the detection of various sulfur molecules in interstellar and, to a lesser extent, outer solar system environments.

## 2 Methodology

**Figure 1:**
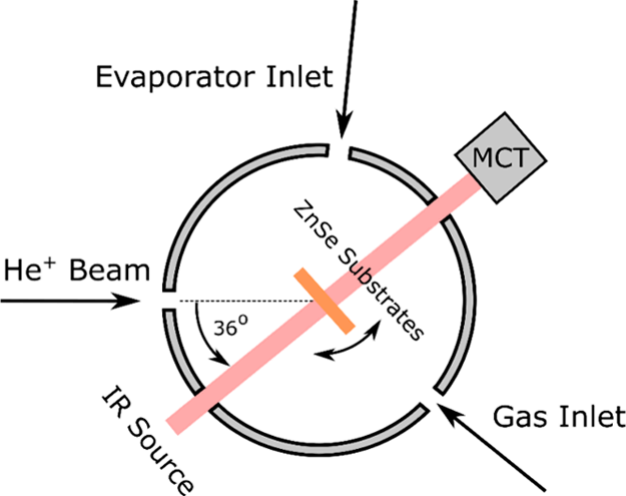
Simplified top-view schematic diagram of the ICA setup. Sulfur layers were prepared onto the cooled ZnSe substrates by rotating them to directly face the nozzle of an effusive evaporator, after which they were rotated back to face an incident mid-infrared spectroscopic beam. Ices were prepared on top of the sulfurous layers through the background condensation of dosed gases and vapors. Irradiation with 1 MeV He^+^ ions was performed using the arrangement shown here, with ions impacting the targets at an angle of 36° to the surface normal. All mid-infrared spectra were acquired in transmission mode by using an external mercury–cadmium–telluride (MCT) detector.

Experiments were carried out using the Ice Chamber for Astrophysics-Astrochemistry (ICA), a laboratory setup for radiation astrochemistry studies located at the HUN-REN Institute for Nuclear Research (Atomki) in Debrecen, Hungary. This setup has been described in detail in previous publications,^54,55^ and so, only the most salient features will be described here. The ICA (Figure 1) is an ultrahigh-vacuum stainless-steel chamber with an operational base pressure of a few 10^–9^ mbar. Within the center of the chamber is a gold-coated, oxygen-free, high-conductivity copper sample holder into which up to four infrared-transparent ZnSe substrates may be mounted. The sample holder and deposition substrates may be cooled to 20 K by virtue of being held in thermal contact with the coldfinger of a closed-cycle helium cryostat; however, the temperature of the sample holder and the substrates may be regulated within the 20–300 K range by establishing an equilibrium between the cooling effect of the cryostat and the warming induced by an internal cartridge heater.

Sulfur-rich interstellar grain analogs were prepared by first cooling the sample holder and substrates down to 20 K (or 70 K if simulating outer solar system objects) and then rotating them to face the nozzle of a commercial effusive evaporator (Createc OLED-40-10-WK-SHM) that had been fitted to the ICA and which had been preloaded with a few grams of pharmaceutical-grade sulfur. The exact allotropic composition of this sulfur is not known, but is likely to be dominated by cyclic S_8_ molecules, due to the known stability of this structure.^56^ The sulfur was subsequently warmed to 78 ± 3 °C and allowed to equilibrate at this temperature for a few minutes, before opening the shutter in front of the evaporator’s nozzle to allow for the deposition of the sulfur on the ZnSe substrates. Sulfur layers were deposited to a thickness of approximately 4 μm (determined through interferometric methods), after which the shutter was closed and the sample holder rotated to face an incident mid-infrared spectroscopic beam. At this point, a background spectrum was acquired to be used as a reference when collecting subsequent mid-infrared absorption spectra.

Interstellar ice analogs composed of neat O_2_, CO, CO_2_, or H_2_O were prepared on top of the sulfur layers by dosing gases or vapors from liquids into the main chamber though a fine regulating needle valve, which allowed for the background deposition of the ices. In the case of the O_2_, CO, and CO_2_ experiments, gases from lecture bottles were used as supplied without any further purification (O_2_ and CO_2_ purity = 99.995%; CO purity = 99.97%; all supplied by Linde), whereas in the case of the H_2_O experiments, vapor was extracted from a deionized water sample that had been degassed through several iterations of the freeze–pump–thaw cycle. The deposition of the interstellar ice analogs could be followed *in situ* using Fourier-Transform mid-infrared transmission absorption spectroscopy over the 4000–650 cm^–1^ range, which allowed for a quantitative assessment of the amount of icy material that was deposited. The column density *N* (molecules cm^–2^) of a deposited ice is related to the integrated absorbance *S* (cm^–1^) of one of its characteristic mid-infrared absorption bands as
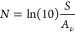
1where *A*_ν_ is the so-called integrated strength constant (cm molecule^–1^) of that band. The constant factor ln(10) is included in eq 1 to allow for the relation of *S*, which is measured on an absorbance scale, to *A*_ν_, which is measured on an optical depth scale. The column density of a deposited ice is related to its thickness *h* (μm) as
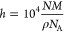
2where *M* is the molar mass of the ice (g mol^–1^), ρ is the ice density (g cm^–3^), and *N*_A_ is the Avogadro constant (6.02 × 10^23^ molecules mol^–1^). The constant 10^4^ is included in eq 2 to express *h* in units of μm.

A list of the integrated band strength constants and densities used to quantify the molecular column densities and thicknesses of the astrophysical ice analogs considered in this study is given in Table 1. Of course, eqs 1 and 2 cannot be used to quantify the column density and thickness of the O_2_ ice, since this species is infrared-inactive due to it being a homonuclear diatomic molecule. Instead, we assumed that the deposition rate of O_2_ is similar to that of CO based on the similarities in the geometries, molecular masses, and chamber pumping speeds of these molecules; and so, depositing an O_2_ ice at the same chamber pressure and for the same duration as was done for the CO ice would result in a similar deposited column density, from which the ice thickness could be determined based on the known density of solid O_2_ (Table 1).

**Table 1:** Absorption Band Strength Constants (*A*_ν_) and Densities (ρ) of the Interstellar Ice Analogs and Sulfur-Rich Grain Analogs Considered in This Study

	**absorption band**	***A*_ν_**	**ρ**
**molecule**	**cm**^**–1**^	**10**^**–17**^cm molecule^**–1**^^a^	**g cm**^**–3**^^b^
S_8_			2.07
O_2_			1.54
CO	2139 (ν_s_)	1.1	0.88
CO_2_	2343 (ν_3_)	7.6	0.98 at 20 K, 1.48 at 70 K
H_2_O	∼3250 (ν_s_)	20.0	0.93

a Integrated band strength constants taken from the work of Gerakines et al.^62^

b Densities of S_8_, O_2_, CO, CO_2_, and H_2_O respectively taken from the works of Greenwood and Earnshaw,^63^ Freiman and Jodl,^64^ Luna et al.,^65^ Satorre et al.,^66^ and Narten et al.^67^

Once deposited to a satisfactory thickness, the interstellar and outer solar system ice analogs prepared on top of sulfur-rich grain analogs were exposed to a 1 MeV He^+^ ion beam supplied by a 2 MV Tandetron accelerator,^57,58^ with incident ions impacting the ices at an angle of 36° to the surface normal. The choice to use 1 MeV He^+^ ions in this study was motivated by two factors: first, such ions are a known component of galactic cosmic rays^59,60^ and are thus relevant to astrochemistry. Second, calculations performed with the *Stopping and Ranging of Ions in Matter* (SRIM) software^61^ revealed that in every case, the penetration range of these ions was less than the combined thickness of the ice and sulfur-rich grain analog (*R*_max_ < 3.4 μm), and thus, the neutralized ions came to rest in the sulfur layer. By making use of helium ions, any chemical modifications of the sulfur layer as a result of its reaction with the implanted particle could be precluded due to the inert nature of the neutral helium atom. Overall, ices were irradiated to a total fluence of 10^15^ ions cm^–2^, with several mid-infrared absorption spectra being acquired at preselected fluence intervals. Spectra were acquired as an average 128 coadded scans and at a resolution of 1 cm^–1^. A summary of the experiments performed is provided in Table 2.

**Table 2:** Summary of the Experiments Performed in This Study, Including Information on the Column Densities (*N*) and Thicknesses (*h*) of the Interstellar Ice Analogs Deposited on Top of Sulfur, as well as the Maximum Penetration Range (*R*_max_) of 1 MeV He^+^ Ions into the Combined Thickness of the Ice and Sulfur-Rich Grain Analog^a^

		***N***	***h***	***R*_max_**
**experiment**	**interstellar ice analog**	**10**^17^ molecules cm^**–2**^	**μm**	**μm**
1	O_2_ at 20 K	5.79	0.20	2.9
2	CO at 20 K	3.78	0.20	3.0
3	CO_2_ at 20 K	0.94	0.07	3.0
4	CO_2_ at 70 K	1.01	0.05	2.9
5	H_2_O at 20 K	37.32	1.20	3.3
6	H_2_O at 70 K	31.10	1.00	3.1

a Note that the temperature at which a particular ice-refractory system was irradiated was the same as the temperature at which it was prepared

## 3 Results and Discussion

### 3.1 O_2_ Ice on Top of Sulfur

**Figure 2:**
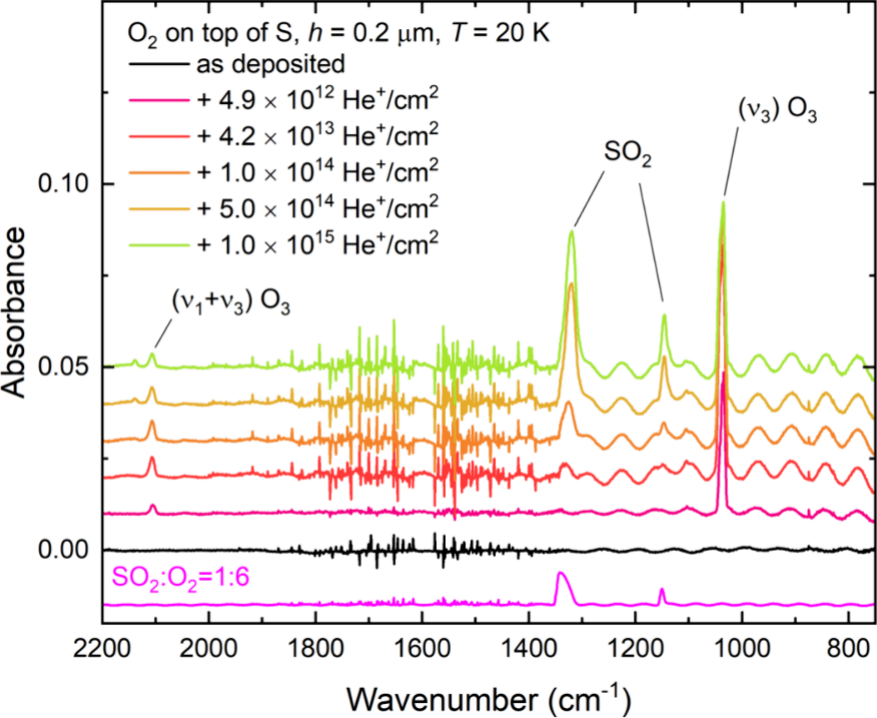
Mid-infrared absorption spectra acquired during the irradiation of a neat O_2_ ice (*h* = 0.2 μm) on top of a layer of allotropic sulfur (*h* ≈ 4 μm) at 20 K using 1 MeV He^+^ ions. A spectrum of pristine, unirradiated (1:6) SO_2_:O_2_ mixed ice is also included for comparison. Spectra are vertically offset from one another in the interest of clarity. Oscillations in the spectra at lower wavenumbers are likely the result of changes in the ice thickness due to ion-induced sputtering of volatile material.

**Figure 3:**
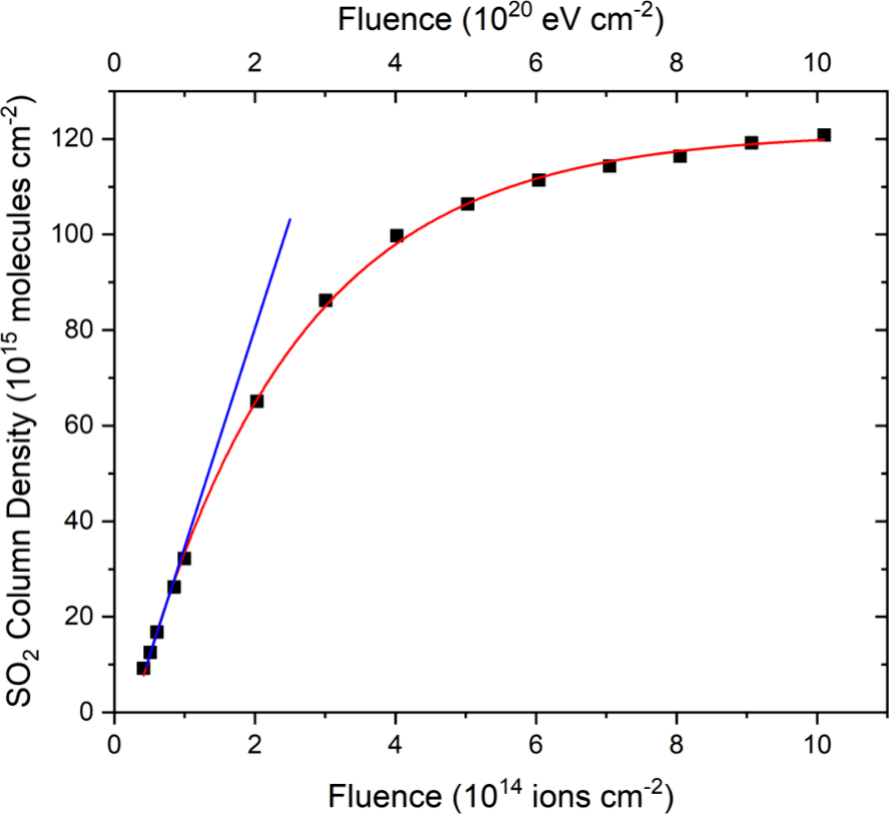
Column density of SO_2_ measured during the irradiation of neat O_2_ ice (*h* = 0.2 μm) on top of a layer of allotropic sulfur (*h* ≈ 4 μm) at 20 K using 1 MeV He^+^ ions. The red curve represents an exponential fit to the data (*r*^2^ > 0.999), while the blue line represents a linear trend (*r*^2^ > 0.999) fitted to the initial points of the exponential curve, from which a *G*-value of 0.0457 ± 0.0004 molecules per 100 eV was calculated.

The irradiation of a neat O_2_ interstellar ice analog on top of a sulfur-rich layer at 20 K resulted in the formation of SO_2_, as was determined by the appearance of two mid-infrared absorption features at about 1335 and 1150 cm^–1^ (Figure 2). The bands have been respectively attributed to the asymmetric and symmetric stretching modes of SO_2_ on the basis of the excellent agreement of their wavenumber positions with those of a reference SO_2_:O_2_ (1:6) ice mixture, as well as their exhibiting the expected SO_2_ spectral profile in which the intensity of the asymmetric stretching mode is greater than that of the symmetric stretching mode.^68^ By making use of an integrated band strength constant of 1.47 × 10^–17^ cm molecule^–1^ for the asymmetric stretching mode,^69^ the column density of SO_2_ could be tracked throughout the irradiation experiment (Figure 3). SO_2_ formation was observed to begin after a fluence of 4.2 × 10^13^ ions cm^–2^ had been delivered, and its measured column density could be fitted by an exponential growth function that plateaued at a value of about 1.2 × 10^16^ molecules cm^–2^. By fitting a linear trend line to the initial points of this exponential growth curve (Figure 3), where the only process likely contributing to the abundance of SO_2_ in the ice is its own radiolytic formation, we could compute a *G*-value of 0.0457 ± 0.0004 molecules per 100 eV.

A number of reactions may contribute to the synthesis of SO_2_ in this experiment and, although it is not possible to confirm with any certainty the dominance of one reaction relative to another, it is possible to speculate on the most likely processes contributing to SO_2_ formation. For instance, it is possible that the reaction of a sulfur allotrope S*_n_* with electronically excited O_2_ could lead to the formation of a cyclic intermediate structure in which one sulfur atom is covalently bonded to the two oxygen atoms, as discussed previously by Mayer.^70^ This sulfur atom then splits off from the intermediate to yield SO_2_ and S_*n–*1_. Although low-temperature solid-phase studies on the addition of atomic or molecular oxygen to allotropic sulfur are generally lacking, previous atmospheric chemistry studies concerned with analogous gas-phase reactions have determined that such reactions are kinetically favorable,^71^ and thus, it is conceivable that they would also proceed under our experimental conditions. Matrix isolation spectroscopic chemiluminescence studies by Long and Pimentel^72^ also demonstrated that electronically excited sulfur atoms may undergo an insertion-type reaction with molecular oxygen to directly yield SO_2_. This reaction is believed to proceed with a near-zero activation energy barrier at temperatures between 10 and 40 K.^72,73^

Oxygen atoms that are formed as a result of the radiolytic dissociation of O_2_ away from the ice-refractory interface may undergo atom addition reactions with O_2_ to yield O_3_. Indeed, both the asymmetric stretching and combination (ν_1_ + ν_3_) modes of O_3_ were detected in acquired mid-infrared absorption spectra of the irradiated ice at 1040 and 2110 cm^–1^,^74^ respectively (Figure 2). The chemistry of O_3_ formation in irradiated ices that are initially rich in O_2_ has been well documented by previous studies,^75−79^ and so will not be discussed in greater detail here.

### 3.2 CO Ice on Top of Sulfur

**Figure 4:**
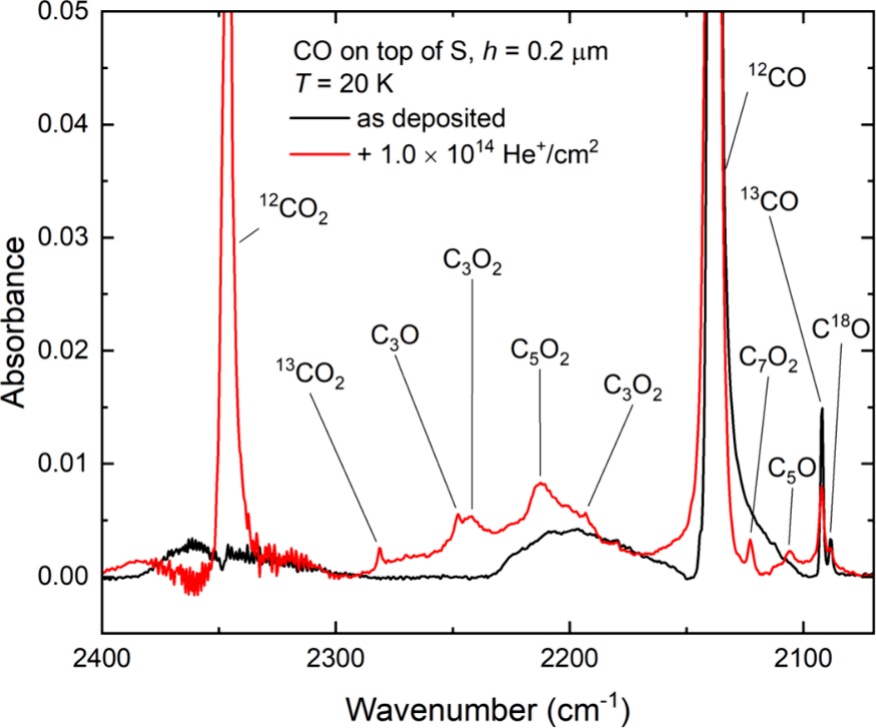
Mid-infrared absorption spectra acquired during the irradiation of neat CO ice (*h* = 0.2 μm) on top of a layer of allotropic sulfur (*h* ≈ 4 μm) at 20 K using 1 MeV He^+^ ions, illustrating the radiolytic synthesis of various oxocarbon molecules in the ice.

**Figure 5:**
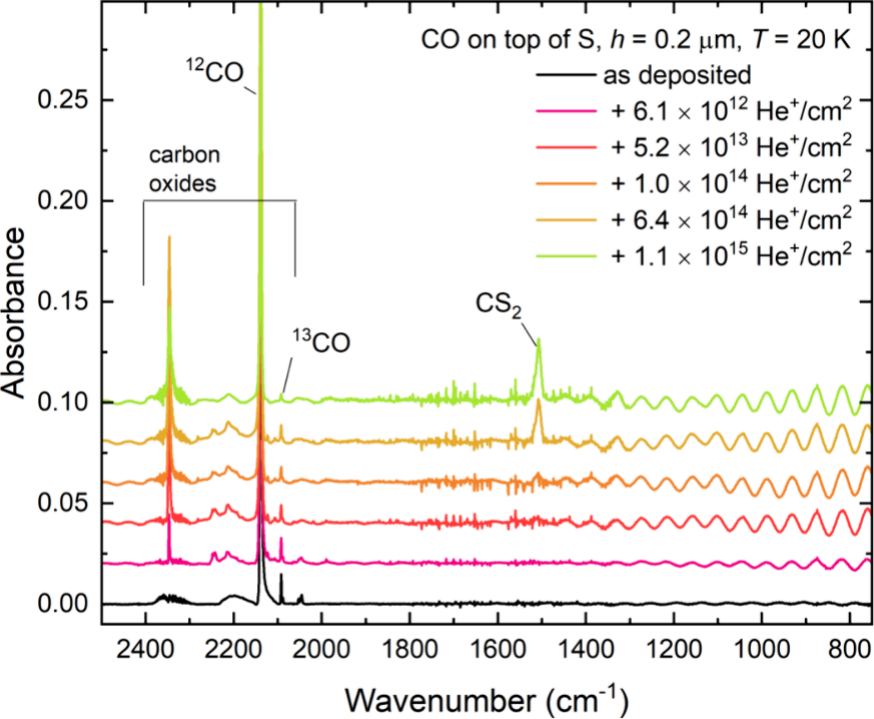
Mid-infrared absorption spectra acquired during the irradiation of a neat CO ice (*h* = 0.2 μm) on top of a layer of allotropic sulfur (*h* ≈ 4 μm) at 20 K using 1 MeV He^+^ ions. Spectra are vertically offset from one another in the interest of clarity. Oscillations in the spectra at lower wavenumbers are likely the result of changes in the ice thickness due to ion-induced sputtering of volatile material.

The irradiation of a CO interstellar ice analog on top of a sulfur-rich layer resulted in the formation of several new products, as evidenced by the appearance of many new absorption features in the acquired mid-infrared absorption spectra (Figures 4 and 5). Many of these new absorption features were clustered in the 2400–2000 cm^–1^ wavenumber range and are associated with the C=O bond stretching modes of various oxocarbon molecules (Table 3).^80−82^ The radiolytic synthesis of these oxocarbon species in irradiated CO-rich interstellar ice analogs has been well documented by previous studies and is believed to proceed through carbon atom addition reactions. In this scheme, electronically excited CO first reacts with ground-state CO to yield CO_2_ and atomic carbon, which may then add to various C*_n_*O species in the ice to yield C_*n*+1_O products.^83,84^ It is also possible for two oxocarbon molecules C*_n_*O and C*_m_*O to combine to yield a cumulene dioxide C*_m+n_*O_2_.^83,84^ It is interesting to note that our results suggest that oxygen atoms are not yielded as part of the mechanistic chemistry occurring during the irradiation of CO ice on top of allotropic sulfur. This is evidenced by the lack of O_3_ or any oxygenated sulfur molecule such as SO_2_ in this experiment, whose synthesis would require the radiolytic liberation and subsequent reaction of oxygen atoms.^71,75−79,85^

**Table 3:** Oxocarbon Molecules Detected after the Irradiation of CO Ice on Top of a Sulfur-Rich Layer at 20 K^a^

**radiolytic product molecule**		
**formula**	**name**	**mid-infrared band position (cm**^**–1**^**)**
^12^CO_2_	carbon dioxide	2346
^13^CO_2_	carbon dioxide	2281
C_3_O	tricarbon monoxide	2247
C_3_O_2_	tricarbon dioxide (carbon suboxide)	2242
C_5_O_2_	pentacarbon dioxide	2211
C_3_O_2_	tricarbon dioxide (carbon suboxide)	2193
^12^CO	carbon monoxide	2138
C_7_O_2_	heptacarbon dioxide	2122
C_5_O	pentacarbon monoxide	2106
^13^CO	carbon monoxide	2092
C^18^O	carbon monoxide	2088

a Band assignments were made on the basis of those made by previous studies.^80−82^

The only detected sulfur-bearing product was CS_2_, which was identified *via* the appearance of its ν_3_ mode at 1509 cm^–1^ (Figure 5).^86^ It should be noted that the exact wavenumber position of this band has been reported to vary somewhat depending on the composition of the molecular ice: for instance, Sivaraman^87^ measured its position to be 1511 cm^–1^ in a mixed CS_2_:CO_2_ ice deposited at 85 K, while Ferrante et al.^46^ measured it to be 1524 cm^–1^ for CS_2_ produced as a result of the irradiation of a H_2_S:CO mixed ice using 800 keV protons at 10 K. The chemical reactions responsible for the synthesis of CS_2_ are somewhat challenging to determine, since literature studies on the formation of this molecule in astrophysical ice analogs are relatively sparse. Ferrante et al.^46^ proposed that CS_2_ could result from the stepwise reaction of CO with two sulfur atoms, which would sequentially yield OCS and OCS_2_. This latter molecule is unstable and thus would dissociate to CS_2_ and atomic oxygen.

However, this reaction mechanism is at odds with the nondetection of the OCS in our experiment (Figure 5). Moreover, the reaction between atomic sulfur and OCS could also conceivably yield CS and S_2_, as discussed by Ikeda et al.,^88^ thereby reducing the formation efficiency of CS_2_. The presence of mobile oxygen atoms within the ice matrix should also have resulted in the synthesis of highly oxygenated products, such as O_3_, which were not detected. Instead, we speculate that an alternative pathway toward CS_2_ may rely on the abundance of free carbon atoms within the irradiated CO ice: these atoms could abstract a sulfur atom from an allotrope of sulfur S*_n_* to yield CS which, being unstable, could then abstract a second sulfur atom to yield the CS_2_ product. To the best of our knowledge, the reaction between carbon atoms and sulfur-containing molecules has not yet been experimentally investigated in the solid phase; however, previous gas-phase work by Deeyamulla and Husain^89^ showed that carbon atoms may abstract sulfur atoms from various simple inorganic sulfur molecules.

**Figure 6:**
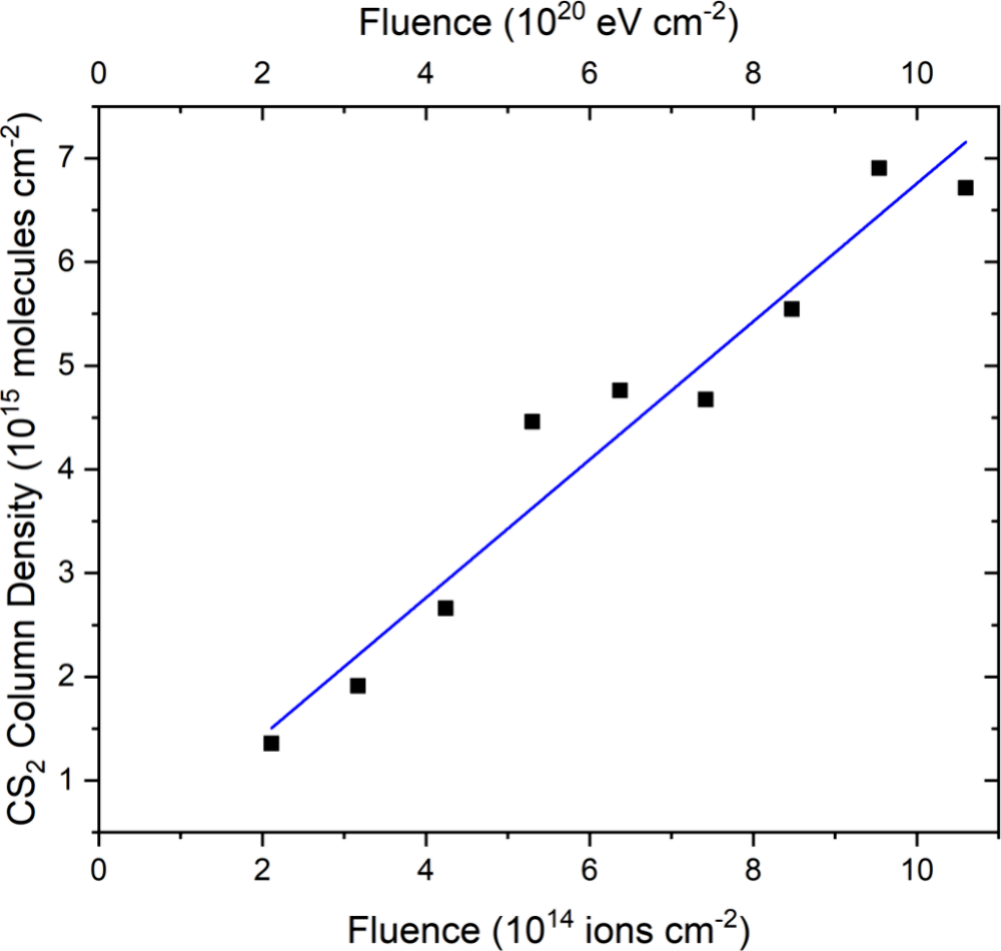
Column density of CS_2_ measured during the irradiation of neat CO ice (*h* = 0.2 μm) on top of a layer of allotropic sulfur (*h* ≈ 4 μm) at 20 K using 1 MeV He^+^ ions. The blue line represents a linear trend fitted to the data (*r*^2^ > 0.94) from which a *G*-value of (6.66 ± 0.50) × 10^–4^ molecules per 100 eV was calculated.

By making use of an integrated band strength constant of 9.1 × 10^–17^ cm molecule^–1^ for the asymmetric stretching mode of CS_2_,^87^ its column density could be quantified as a function of fluence. As can be seen in Figure 5, CS_2_ is unambiguously detectable in acquired mid-infrared spectra at fluences greater than 10^14^ ions cm^–2^, and its column density was found to grow in a linear fashion with increasing ion fluence (Figure 6). The overall abundance of CS_2_ yielded is low when compared to the abundance of SO_2_ produced during the analogous irradiation of O_2_ ice on allotropic sulfur (Figure 3). As such, it is likely that the data in Figure 6 represent the earliest fluence points at which CS_2_ synthesis occurs within the irradiated ice, and at which any other chemical processes that could impact the abundance of CS_2_ within the ice are negligible. Therefore, a linear trend line could be fitted to the entire data set so as to calculate a *G*-value of (6.66 ± 0.50) × 10^–4^ molecules per 100 eV.

### 3.3 CO_2_ Ice on Top of Sulfur

**Figure 7:**
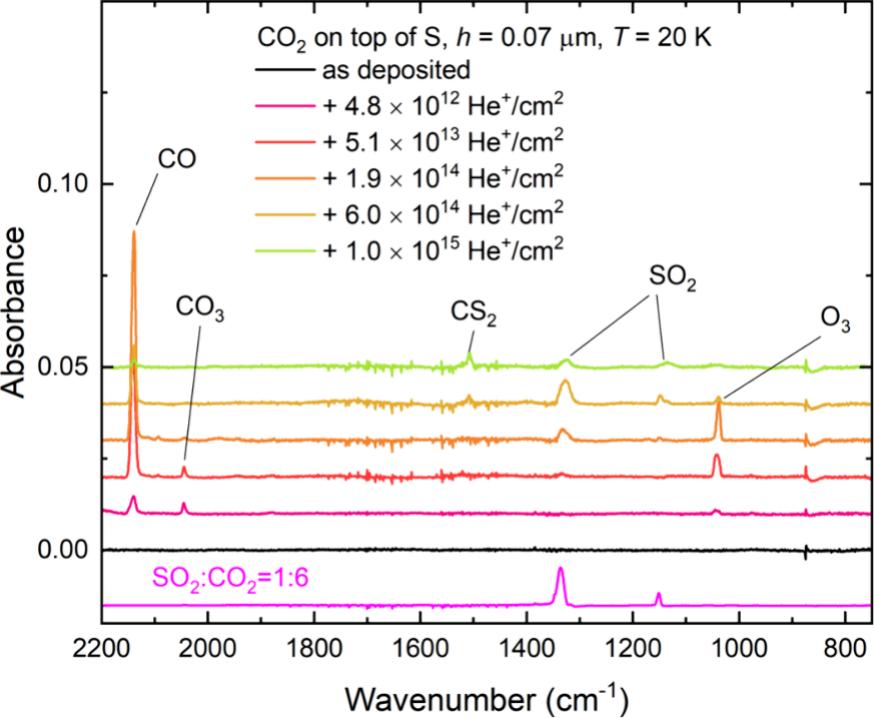
Mid-infrared absorption spectra acquired during the irradiation of neat CO_2_ ice (*h* = 0.07 μm) on top of a layer of allotropic sulfur (*h* ≈ 4 μm) at 20 K using 1 MeV He^+^ ions. Spectra are vertically offset from one another in the interest of clarity.

The irradiation of CO_2_ ice on top of allotropic sulfur was performed at two temperatures (i.e., 20 and 70 K) to allow for a comparison of the possible chemistry occurring within dense molecular clouds in the interstellar medium to that on the surfaces of cold outer solar system objects. At 20 K, the irradiation resulted in the formation of a number of new product molecules (Figure 7), including those known to be yielded as a result of the radiolytic processing of solid CO_2_, such as CO, CO_3_, and O_3_.^90−93^ Moreover, the absorption features of both SO_2_ and CS_2_ were detected in mid-infrared spectra acquired during irradiation. The radiation chemistry leading to the formation of all of these molecules is very rich, and multiple reaction pathways may contribute to the formation of common molecules. The irradiation of CO_2_ results in the homolytic dissociation of one C=O bond, yielding CO and atomic oxygen as radiolytic products.^94^ The addition of this oxygen atom to a parent CO_2_ molecule may yield the CO_3_ product, while the combination of free oxygen atoms may sequentially yield O_2_ and O_3_.^90^

We propose that there are at least three reaction pathways through which SO_2_ can be produced. Two of these routes are identical to those proposed to account for the formation of SO_2_ as a result of the irradiation of O_2_ ice on top of allotropic sulfur, namely, the insertion-type reaction of electronically excited sulfur atoms into O_2_ molecules generated *via* the combination of radiolytically derived oxygen atoms, and the addition of O_2_ to allotropic sulfur S*_n_* to yield a cyclic intermediate that decomposes to yield SO_2_ and S_*n*–1_. The third possible route involves the reaction of CO_2_ with a sulfur atom to yield CO and SO,^95^ prior to the oxidation of the latter species to yield SO_2_.^71,85^ On the other hand, the most likely reaction sequence leading to the formation of CS_2_ is analogous to that proposed by Hawkins et al.^96^ and Ferrante et al.,^46^ in which CO sequentially abstracts two atoms of sulfur from an allotropic structure to yield OCS and OCS_2_, after which this latter species dissociates to CS_2_ and atomic oxygen.^97^ However, as was demonstrated by Ferrante et al.,^46^ OCS is not particularly stable when processed by ionizing radiation and therefore may not accumulate within the ice, hence explaining its nondetection in the experiment performed at 20 K. Aside from the reaction with sulfur to yield OCS_2_,^46,96^ OCS may react with atomic oxygen to yield CO and SO,^98−100^ thus further contributing to the production of SO_2_*via* the rapid oxidation of the SO intermediate.^71,85^ It should also be noted that OCS_2_ need not necessarily dissociate to CS_2_ and atomic oxygen, but may also dissociate to CO and S_2_.^101^ Although S_2_ is a volatile sulfur allotrope,^102^ and thus represents one outcome of the volatilization of the deposited sulfur layer, its formation could not be confirmed through mid-infrared spectroscopy due to it being a homonuclear diatomic molecule and thus infrared-inactive.

**Figure 8:**
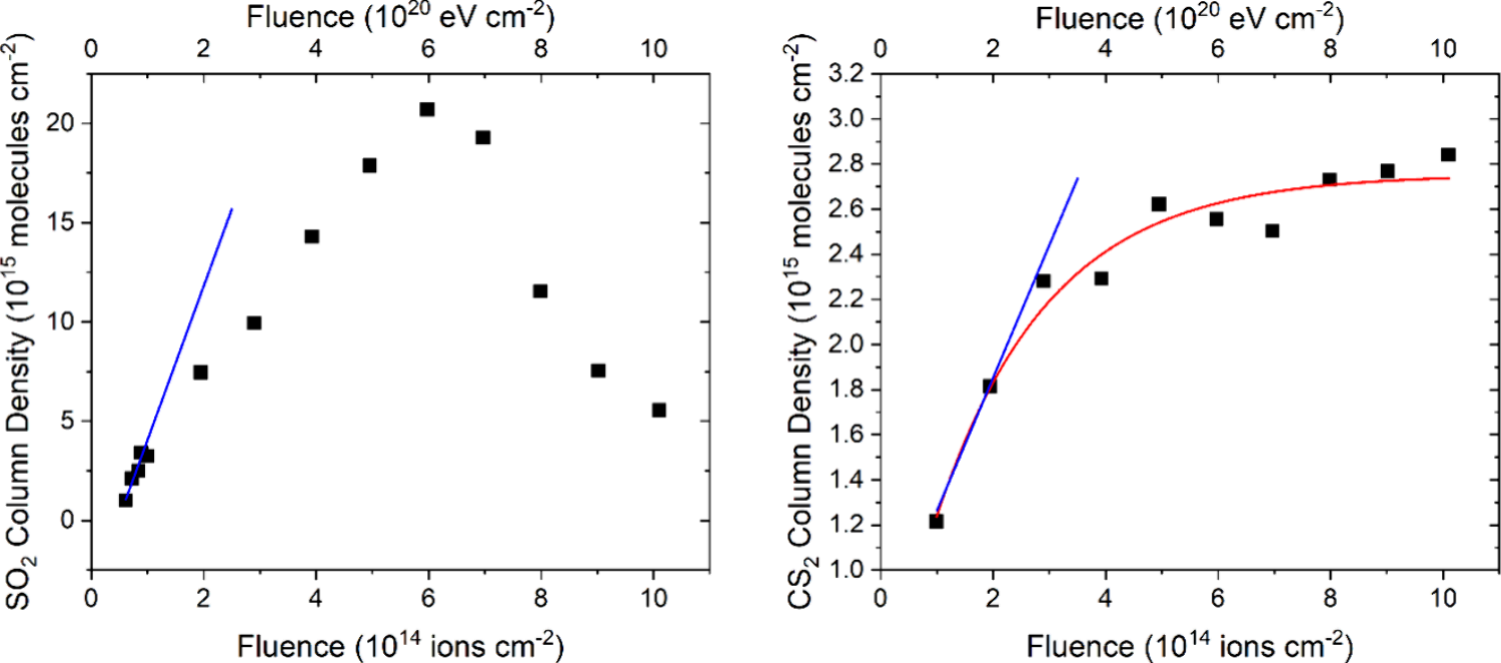
Column density of SO_2_ (left panel) and CS_2_ (right panel) measured during the irradiation of neat CO_2_ ice (*h =* 0.07 μm) on top of a layer of allotropic sulfur (*h* ≈ 4 μm) at 20 K using 1 MeV He^+^ ions. Note than an exponential curve could not be fitted to the SO_2_ data, and thus, the blue line represents a linear trend fitted to the first four data points (*r*^2^ > 0.90) from which a *G*-value of 0.0078 ± 0.0014 molecules per 100 eV was calculated. Conversely, an exponential curve could be fitted to the CS_2_ data (red curve; *r*^2^ > 0.95), to which a linear trend (blue line; *r*^2^ > 0.99) could be fitted to its initial points. From this linear trend, a *G*-value of (5.89 ± 0.13) × 10^–4^ molecules per 100 eV was calculated.

The column densities of both SO_2_ and CS_2_ were measured as a function of fluence throughout the 20 K experiment (Figure 8). Interestingly, the column density of SO_2_ steadily increased until a fluence of 6 × 10^14^ ions cm^–2^ had been delivered, after which point it began to decrease. Such a trend was also observed for the non-sulfur-bearing radiolytic products, whose absorption bands all began to decrease in intensity at higher fluences (Figure 7). CS_2_ was first observed in the irradiated ice after a fluence of about 10^14^ ions cm^–2^ had been delivered and, in contrast to the trends observed for the other products of this irradiation, its column density steadily increased before plateauing at about 2.7 × 10^15^ molecules cm^–2^ by the end of the experiment (Figure 8). *G*-values for the SO_2_ and CS_2_ products were calculated to be 0.0078 ± 0.0014 and (5.89 ± 0.13)×10^–4^ molecules per 100 eV, respectively. The reason for the apparent decline in the column densities of all radiolytic products other than CS_2_ at higher fluences is difficult to explain; however, a likely contributor to this observation was ice sputtering, which has been described in detail for CO_2_ ices bombarded by ions at low temperatures (i.e., <25 K).^103^ It is also possible to speculate that the greater relative abundances of CS_2_ within the ice at higher fluences allowed for the consumption of volatile ice components in the formation of carbon–sulfur polymers that could not be detected using mid-infrared absorption spectroscopy. Indeed, prior research has documented the efficient formation of such complex polymeric structures after the irradiation of CS_2_ and that these polymers exhibit very weak or difficult-to-observe absorptions over mid-infrared wavelengths.^104−106^

**Figure 9:**
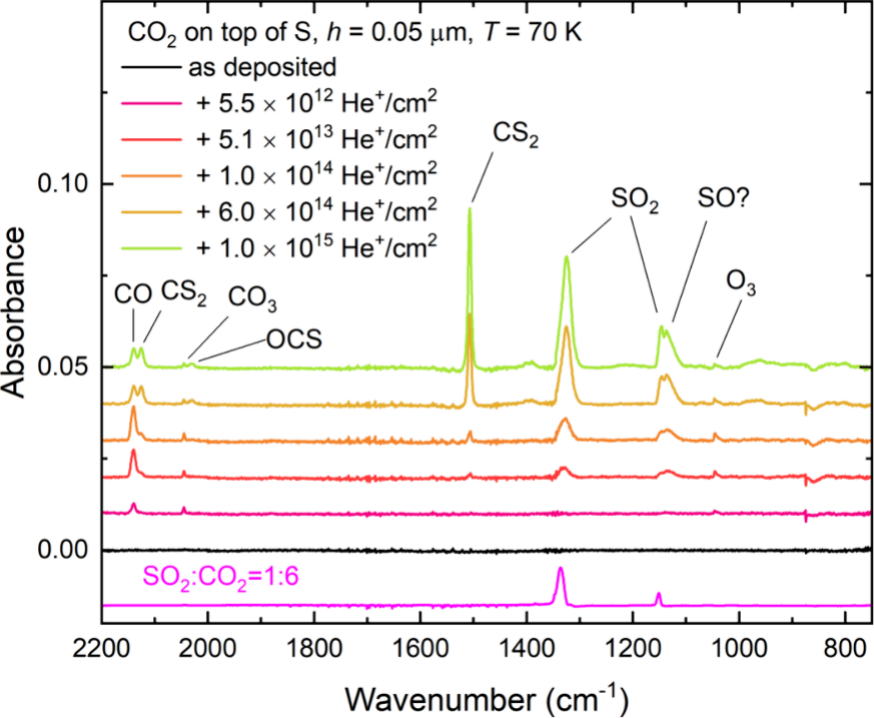
Mid-infrared absorption spectra acquired during the irradiation of neat CO_2_ ice (*h* = 0.05 μm) on top of a layer of allotropic sulfur (*h* ≈ 4 μm) at 70 K using 1 MeV He^+^ ions. Spectra are vertically offset from one another in the interest of clarity.

**Figure 10:**
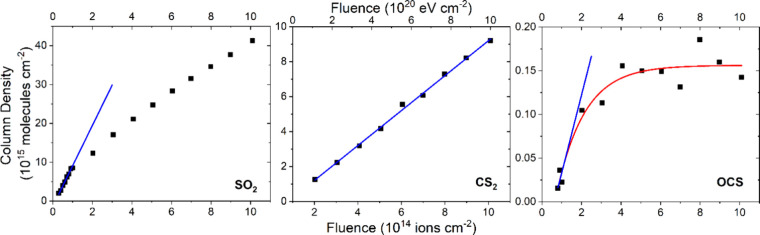
Column density of SO_2_ (left panel), CS_2_ (center panel), and OCS (right panel) measured during the irradiation of neat CO_2_ ice (*h* = 0.05 μm) on top of a layer of allotropic sulfur (*h* ≈ 4 μm) at 70 K using 1 MeV He^+^ ions. Note that a *G*-value of 0.0104 ± 0.0003 molecules per 100 eV was calculated for SO_2_ by fitting a linear trend line (blue line; *r*^2^ > 0.99) to the initial seven data points. A *G*-value of (9.99 ± 0.19) × 10^–4^ molecules per 100 eV was calculated for CS_2_ by fitting a linear trend line (blue line; *r*^2^ > 0.997) to the entire data set. Lastly, an exponential curve could be fitted to the OCS data (red curve; *r*^2^ > 0.92), to which a linear trend line (blue line; *r*^2^ > 0.999) could be fitted to its initial points. From this linear trend, a *G*-value of (0.89 ± 0.01) × 10^–4^ molecules per 100 eV was calculated.

The spectroscopic results of the 1 MeV He^+^ ion irradiation of CO_2_ ice on top of sulfur at 20 K (Figure 7) contrast strongly with those of the analogous experiment performed at 70 K (Figure 9). Perhaps the most evident difference is that at this higher temperature, the irradiation of the ice results in higher abundances of the sulfur-bearing products SO_2_ and CS_2_, whose column densities were found to increase with higher fluences (Figure 10). Moreover, OCS was detected through the appearance of a broad absorption band centered at 2031 cm^–1^ in acquired mid-infrared spectra (Figure 9), attributable to its ν_3_ mode. The position of the peak of this broad band is known to shift by up to 30 cm^–1^ based upon the composition of the ice;^23,46,87^ however, our observation is in good agreement with that reported by Sivaraman,^87^ who observed it at 2035 cm^–1^ after the irradiation of a CO_2_:CS_2_ mixed ice at 85 K, and is in reasonable agreement with that reported by Ferrante et al.,^46^ who observed it at 2019 cm^–1^ for a neat OCS ice prepared at 10 K and subsequently warmed to 25 K. It is to be further noted that the position of this OCS absorption feature is in close proximity to the ν_1_ mode of CO_3_; however, these two bands may be distinguished from each other on the basis of their full-widths at half-maxima,^46^ since the CO_3_ ν_1_ mode is known to present as a relatively narrow band. Indeed, in our experiment, the OCS absorption band at 2031 cm^–1^ had a significantly larger full-width at half-maximum of 11 cm^–1^, compared to the more modest 4 cm^–1^ for the CO_3_ band at 2045 cm^–1^ (Figure 9).

Interestingly, although the irradiation of CO_2_ ice on top of sulfur at 70 K led to higher abundances of sulfur-bearing products (i.e., SO_2_, CS_2_, and OCS) compared to the analogous experiment at 20 K, abundances of nonsulfur-bearing products (i.e., CO, CO_3_, and O_3_) were generally lower (Figures 7 and 9). Given that the initial abundances of both CO_2_ ice and allotropic sulfur were similar across both experiments (Table 2), the difference in these results must stem from the different reaction pathways being favored at these two temperatures. Since radicals are known to be more mobile within solid matrices at higher temperatures, it is likely that large sulfur atoms released from the underlying layer of allotropic sulfur as a result of its irradiation can migrate further into the overlying ice layer at 70 K than they could have done at 20 K. Indeed, the increased mobility of sulfur atoms in an ice matrix at higher temperatures has already been demonstrated in previous studies.^107,108^ Therefore, the comparatively higher concentration of sulfur atoms intermixed with CO_2_ ice could conceivably promote the reaction leading to SO and CO, which then produces SO_2_ upon oxidation of the SO.^71,85^ Some tentative evidence for the enhancement of this reaction lies in the appearance of the SO_2_ symmetric stretching mode which, unlike in the analogous 20 K experiment, appears to be split into a double-peaked structure with peaks at 1046 and 1036 cm^–1^ (Figure 9). We speculate that this double-peaked structure is the result of contributions from the symmetric stretching modes of SO_2_ (at 1046 cm^–1^) and of SO (at 1036 cm^–1^).^97,109^ Although SO is generally an unstable species that is typically rapidly oxidized to SO_2_, it is possible that the productivity of the sulfur chemistry occurring in our 70 K irradiation experiment allows for the sustained production of this radical and thus its tentative spectroscopic detection. Moreover, the higher concentration of sulfur atoms within the irradiated CO_2_ ice would also compete more efficiently for oxygen atoms and CO molecules, which would have the net effect of reducing the abundance of these species and, by extension, of both O_3_ and CO_3_ within the irradiated ice.

The column densities of SO_2_, CS_2_, and OCS were tracked throughout the 70 K irradiation experiment (Figure 10). In the case of SO_2_, it was apparent that the column density initially increased linearly as a function of fluence; however, beginning at a fluence of approximately 9 × 10^13^ ions cm^–2^, the gradient of this linear trend decreased. As such, the *G*-value, which was found to be 0.0104 ± 0.0003 molecules per 100 eV, was calculated by fitting a trend line to the initial higher gradient linear data points at the earliest fluences. The evolution of the CS_2_ column density was linear throughout the irradiation experiment (Figure 10) and, indeed, was qualitatively similar to the accumulation of CS_2_ observed during the irradiation of neat CO ice on top of sulfur at 20 K (Figure 6). The *G*-value for this product, which was calculated to be (9.99 ± 0.19) × 10^–4^ molecules per 100 eV, was therefore found by fitting a linear trend line to the entire data set. Finally, the column density of OCS was measured by making use of an integrated band strength constant of 1.5 × 10^–16^ cm molecule^–1^ for the asymmetric stretching mode^110^ and was found to follow a general exponential growth trend, albeit at low abundances (Figure 10). This is perhaps reflective of the comparative radiolytic instability of the OCS, which precludes it from accumulating within the ice during irradiation. By fitting a linear trend line to the initial points of this exponential growth curve, where radiolytic formation is likely the only process contributing to the abundance of OCS, a *G*-value of (0.89 ± 0.01) × 10^–4^ molecules per 100 eV could be calculated.

### 3.4 H_2_O Ice on Top of Sulfur

**Figure 11:**
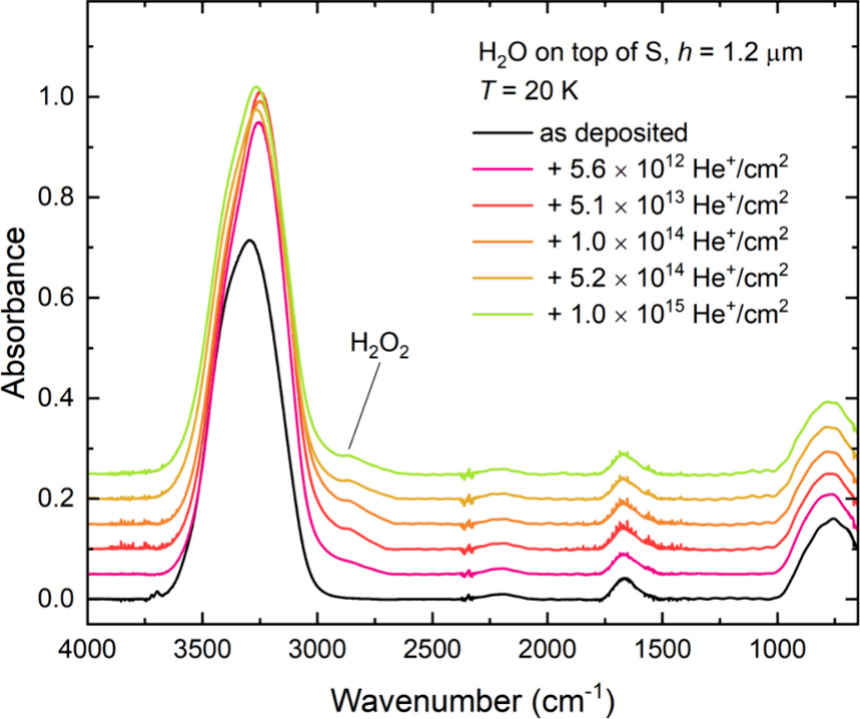
Mid-infrared absorption spectra acquired during the irradiation of neat H_2_O ice (*h* = 1.2 μm) on top of a layer of allotropic sulfur (*h* ≈ 4 μm) at 20 K using 1 MeV He^+^ ions. Spectra are vertically offset from one another in the interest of clarity.

The mid-infrared spectra acquired during the irradiation of H_2_O ice on top of allotropic sulfur at 20 K are shown in Figure 11. It is possible to note that irradiation does not result in many noticeable changes to the appearance of the spectra, other than the emergence of a weak but broad absorption band centered at 2863 cm^–1^, which has been attributed to the formation of H_2_O_2_.^111−114^ Furthermore, two small absorption features at 3696 and 3721 cm^–1^ that were apparent in the mid-infrared spectrum of the H_2_O ice upon its preparation at 20 K were observed to disappear very rapidly during irradiation. These bands were attributed to the OH dangling bonds that exist in microporous H_2_O ice at low temperatures, and their disappearance is evidence of the ice having undergone a process of radiation-induced compaction.^115−117^ A particularly interesting result of this experiment is the fact that no sulfur-bearing products could be identified during irradiation. Comparisons with the results of previous studies are somewhat challenging, since most previous experimental work was performed with the aim of characterizing radiation-induced sulfur chemistry on the surfaces of the icy Galilean moons of Jupiter and thus was carried out at higher temperatures.

Indeed, to the best of our knowledge, the only experiment performed at a comparable temperature to that of the present experiment is that of Kaňuchová et al.,^118^ who investigated the 30 keV He^+^ ion irradiation of a mixed H_2_O:SO_2_ (1:2) ice at 16 K. Their work demonstrated that the irradiation of this ice resulted in the formation of H_2_SO_4_ and its monohydrate and tetrahydrate forms, as inferred from mid-infrared absorption bands at 1105 and 1070 cm^–1^ that were respectively attributed to the presence of HSO_4_^–^ and SO_4_^2–^ ions, as well as the broad H_3_O^+^ band centered at about 1730 cm^–1^. This result contrasts strongly with that presented in Figure 11, where no such mid-infrared absorption bands could be identified. The reason for this difference may, however, be fairly mundane: it is likely that the sulfur chemistry occurring in the experiment of Kaňuchová et al.^118^ occurred to a greater extent due to the more homogeneous mixing of the SO_2_ and H_2_O components of their ice, thus allowing for sulfur product formation to occur uniformly throughout the ice. This was not the case in the present experiment, where the radiolytic formation of sulfur-bearing products could only occur at the ice-refractory interface and its immediate environs and was likely limited by the low mobilities of sulfur atoms at 20 K.^107,108^

**Figure 12:**
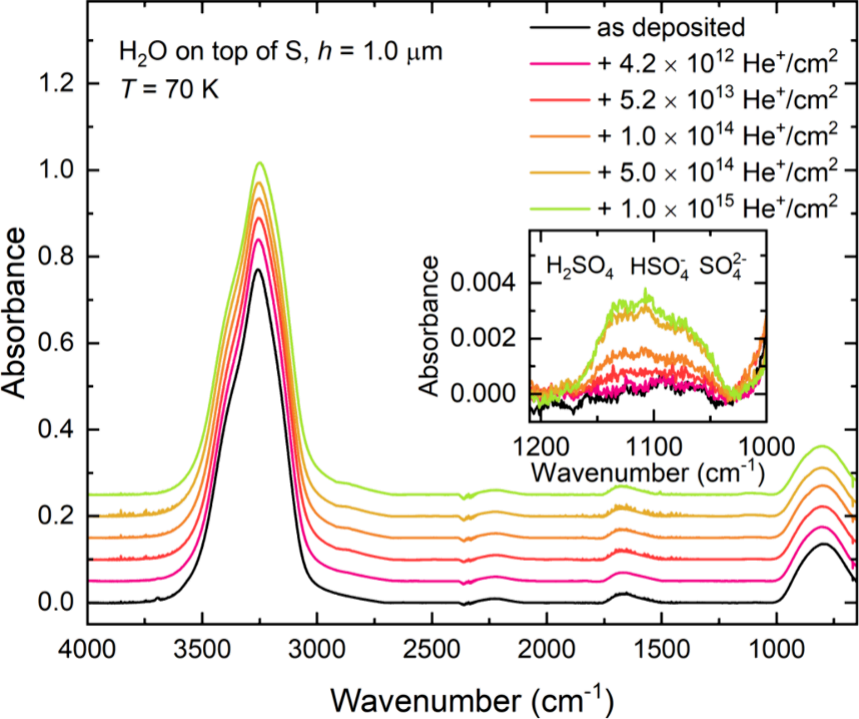
Mid-infrared absorption spectra acquired during the irradiation of neat H_2_O ice (*h* = 1.0 μm) on top of a layer of allotropic sulfur (*h* ≈ 4 μm) at 70 K using 1 MeV He^+^ ions. Spectra are vertically offset from one another in the interest of clarity.

The 1 MeV He^+^ ion irradiation of H_2_O ice on top of a layer of allotropic sulfur was also performed at 70 K, and, in this case, spectroscopic evidence for the radiation-induced formation of H_2_SO_4_ and its monohydrate and tetrahydrate forms could be identified (Figure 12). In particular, absorption features at 1139, 1107, and 1060 cm^–1^ were observed and could be respectively associated with the formation of H_2_SO_4_, HSO_4_^–^, and SO_4_^2–^ within the irradiated ice.^118−120^ Although the microporous structure of H_2_O ice, which has been previously described as a facilitator of radical and molecule mobility throughout the ice matrix, is known to vary with ice temperature,^115−117,121,122^ we do not believe that this was the main driver behind the difference in the chemical productivities of our experiments conducted at 20 and 70 K. This is due to the fact that absorption bands associated with OH dangling bonds were initially detected in spectra of the H_2_O ices acquired before irradiation at both these temperatures, but quickly disappeared during irradiation due to ice compaction and micropore destruction, even as the abundances of the acid hydrate products continued to increase. Moreover, bands associated with the OH dangling bonds were more intense in the 20 K ice compared with the 70 K ice, indicating a reduced porosity in the ice prepared at the higher temperature. Nevertheless, more extensive sulfur chemistry was observed in the H_2_O ice irradiated on top of sulfur at 70 K, thus indicating that the dominant driver of this chemistry is more likely to be the greater thermally driven mobility of radicals and molecules in the 70 K ice; particularly of large sulfur atoms released from the underlying layer of allotropic sulfur which would then migrate further into the ice structure and trigger more extensive chemistry at this higher temperature.^107,108^ A similar argument was made to explain the enhanced formation of sulfur-bearing products as a result of the irradiation of CO_2_ ice on top of sulfur at 70 K compared with 20 K.

**Scheme 1:**
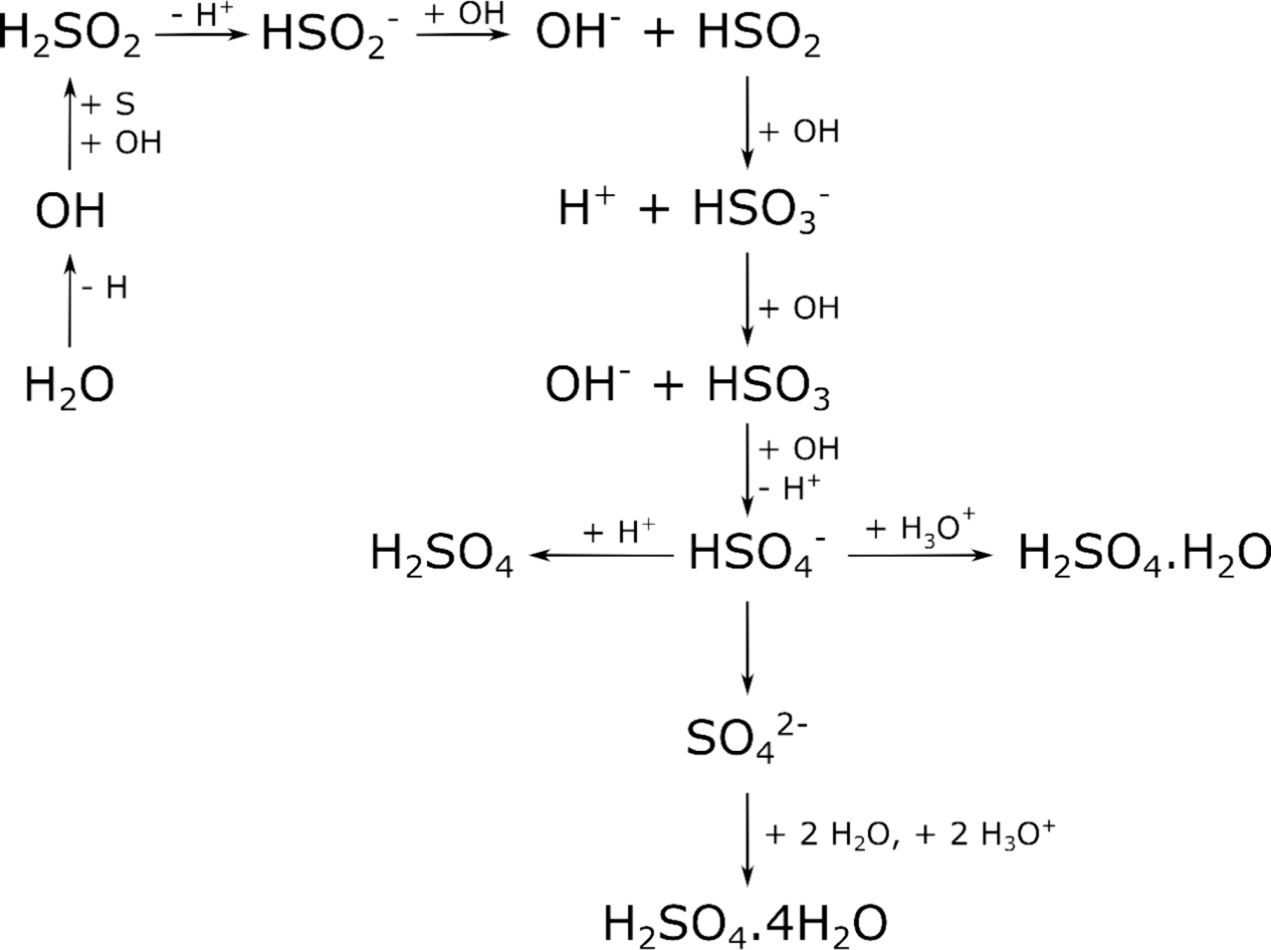
Reaction Network Proposed by Della Guardia and Johnston^123^ Leading to the Formation of H_2_SO_4_ and Its Monohydrate and Tetrahydrate Forms after the Irradiation of H_2_O Ice on Top of Sulfur at 70 K Using 1 MeV He^+^ Ions

The reactions leading to the formation of these acid hydrate products were described in detail by Della Guardia and Johnston,^123^ who highlighted the important role played by OH radicals and H_2_SO_2_, a lower oxoacid of sulfur whose astrochemical importance was expounded upon more recently by Góbi et al.^124^ In this reaction network (shown in Scheme 1), OH radicals produced from the radiolytic dissociation of H_2_O react with atomic sulfur to yield H_2_SO_2_, which then dissociates to H^+^ and HSO_2_^–^. The anion is subsequently neutralized *via* a charge exchange reaction with OH, before a subsequent reaction with OH oxidizes it to HSO_3_^–^. Another round of neutralization *via* charge exchange followed by oxidation mediated by the reaction with OH radicals yields HSO_4_^–^. At this point, HSO_4_^–^ may react in three different ways to yield H_2_SO_4_, the monohydrate acid, or the tetrahydrate acid. The H_2_SO_4_ product is formed as a result of the simple combination reaction between H^+^ and HSO_4_^–^, while the monohydrate is formed *via* the combination reaction between H_3_O^+^ and HSO_4_^–^. Finally, the tetrahydrate is produced in a two-step process in which HSO_4_^–^ first dissociates to H^+^ and SO_4_^2–^, after which two H_3_O^+^ ions and two neutral H_2_O molecules associate with the SO_4_^2–^ anion.

**Figure 13:**
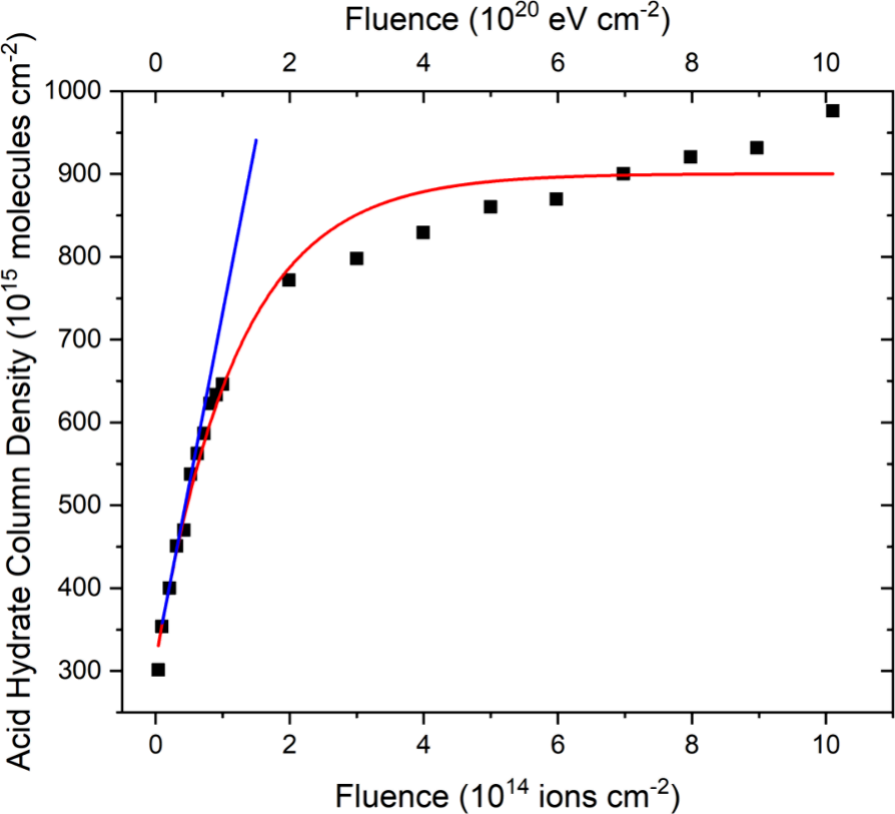
Total column density of acid hydrates (i.e., H_2_SO_4_·*n*H_2_O; *n* = 0, 1, 4) measured during the irradiation of neat H_2_O ice (*h* = 1.0 μm) on top of a layer of allotropic sulfur (*h* ≈ 4 μm) at 70 K using 1 MeV He^+^ ions. The red curve represents an exponential fit to the data (*r*^2^ > 0.97), while the blue line represents a linear trend (*r*^2^ > 0.999) fitted to the initial points of the exponential curve, from which a *G*-value of 0.4163 ± 0.0031 molecules per 100 eV was calculated.

The integrated absorbances of the bands related to the acid hydrate products were not easy to measure in the mid-infrared spectra acquired during irradiation, and thus, we have made use of so-called difference spectra in order to facilitate these measurements. Difference spectra were produced by subtracting the spectrum acquired at zero fluence from all spectra subsequently acquired during irradiation, as shown in the inset of Figure 12. Furthermore, in measuring the column densities of the acid hydrate products, we have followed the example of Ding et al.^125^ and have integrated over all acid hydrate absorption bands across the 1350–760 cm^–1^ wavenumber range, making use of a band strength constant of 1.3 × 10^–17^ cm molecule^–1^. The total abundance of the acid hydrate products increased in an exponential-like fashion (Figure 13), and by fitting a linear trend line to the initial points of the fitted curve, a *G*-value of 0.4163 ± 0.0031 molecules per 100 eV could be calculated.

## 4 Astrochemical Implications and Conclusions

In this study, we investigated whether simple inorganic sulfur-bearing molecules that could be detected by ground- or space-borne telescopes can be formed in interstellar ices (and, to a lesser extent, outer solar system ices) as a result of the irradiation of sulfur-free icy material on top of sulfur-rich dust grains. Our experiments have demonstrated that several such molecules can be formed in this way, including SO_2_, CS_2_, OCS, and H_2_SO_4_ hydrates. One particularly noteworthy result of our experiments performed with H_2_O ice was the nondetection of H_2_S among the radiolytic products. Indeed, the identification of this molecule in interstellar icy grain mantles has thus far also eluded observational astronomers. This is perhaps counterintuitive, as surface-catalyzed hydrogen atom addition reactions are known to be a feasible route toward the formation of simple molecules in dense molecular clouds,^126^ and so the hydrogenation of grain-adsorbed sulfur atoms could in principle offer a route toward interstellar H_2_S. Although past theoretical studies have suggested that this hydrogenation reaction is accompanied by very high energy barriers when sulfur is adsorbed on a platinum surface,^127^ it is important to note that, to the best of our knowledge, this reaction route has not been studied either theoretically or experimentally using surfaces that are more relevant to astrochemistry and so its possible contribution to interstellar H_2_S is unknown. Recent studies have demonstrated that H_2_S is very likely to undergo chemical desorption (also sometimes referred to as “reactive desorption”) as a result of its formation *via* hydrogen atom addition to HS radicals,^128,129^ which may account for its nondetection in interstellar icy grain mantles but its positive detection in the gas-phase within interstellar media.^130−132^ In any case, our results suggest that the possible formation and retention of H_2_S in the ice phase as a result of the cosmic ray or stellar wind irradiation of H_2_O ice on allotropic sulfur is not efficient, and thus, alternative strategies to explain its hypothesized presence in icy astrophysical environments must be sought.

**Figure 14:**
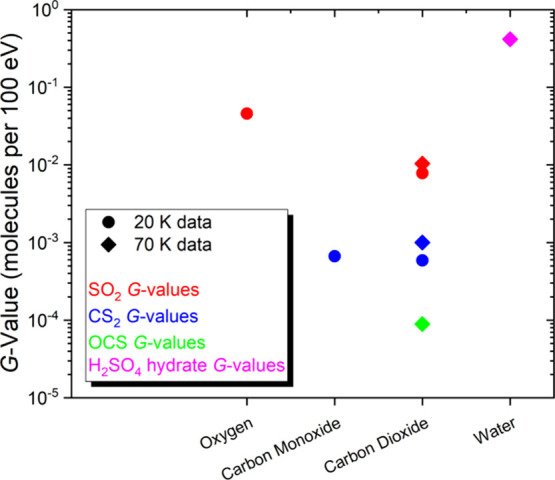
Calculated *G*-values for the sulfur-bearing products observed as a result of irradiating different ices on top of allotropic sulfur using 1 MeV He^+^ ions. Note that circles represent data points acquired at 20 K, while diamonds represent those acquired at 70 K. Information about error bars can be found in Table 4.

As can be noted in Figure 14 and Table 4, the most commonly observed sulfur-bearing products in our experiments were SO_2_ and CS_2_. Comparing the *G*-values of these molecules, it is possible to note two distinct trends: first, SO_2_ is in general more readily produced than CS_2_ as demonstrated by its higher *G*-values, and second, the irradiation of a given ice on top of sulfur at 70 K is more productive than the analogous irradiation at 20 K, most likely due to the higher mobilities of radiolytically derived radicals at higher temperatures. Given that SO_2_ and CS_2_ were observed in experiments performed at both 20 and 70 K, our results therefore indicate that the irradiation of volatile ices on top of allotropic sulfur is a plausible strategy toward the synthesis of these molecules in both interstellar icy grain mantles as well as on the surfaces of icy outer solar system bodies. Therefore, it may be worthwhile for future observational studies to search for spectroscopic evidence of CS_2_ in astrophysical ices, such as in interstellar icy grain mantles where it may coexist alongside the already detected SO_2_.^11,12^

**Table 4:** *G*-Values Calculated for the Sulfur-Bearing Products Observed as a Result of Irradiating Different Neat Ices on Top of Allotropic Sulfur Using 1 MeV He^+^ Ions

	*G***-values (molecules per 100 eV) for sulfur products**			
**ice**	**SO**_**2**_	**CS**_**2**_	**OCS**	**H**_**2**_**SO**_**4**_**hydrates**
O_2_ ice at 20 K	0.0457 ± 0.0004			
CO ice at 20 K		(6.66 ± 0.50) × 10^–4^		
CO_2_ ice at 20 K	0.0078 ± 0.0014	(5.89 ± 0.13) × 10^–4^		
CO_2_ ice at 70 K	0.0104 ± 0.0003	(9.99 ± 0.19) × 10^–4^	(0.89 ± 0.01)×10^–4^	
H_2_O ice at 20 K				
H_2_O ice at 70 K				0.4163 ± 0.0031

Another result of interest was the comparatively low *G*-value of OCS, which was only detected after the irradiation of CO_2_ ice on sulfur at 70 K. The fact that OCS could not be detected in our experiments conducted at the more interstellar-relevant temperature of 20 K suggests that the cosmic ray irradiation of volatile ices on top of allotropic sulfur is not a major contributor toward the formation of this species, strongly contrasting with the apparent ease with which SO_2_ is formed in this way. This result complements the recent observational surveys of SO_2_ and OCS toward protostellar regions carried out by Santos et al.,^133^ who measured a weak correlation between the gas-phase column densities of these two sulfur-bearing molecules. This was interpreted as the result of these two molecules having different formation chemistries during distinct interstellar cloud evolutionary phases. Indeed, in that and subsequent studies, Santos et al.^133,134^ proposed that solid SO_2_ is likely formed as a result of the energetic processing (e.g., by galactic cosmic rays) during the low-density phase of interstellar molecular clouds when the surface-catalyzed formation of oxygen-rich molecules (e.g., H_2_O, O_2_, and CO_2_) is a dominant process.^135,136^ Conversely, the formation of OCS was proposed to occur after the catastrophic freeze-out of gas-phase CO as a result of a neutral–neutral reaction between CO molecules and HS radicals to yield HSCO as an intermediate species, which, upon hydrogenation, dissociates to OCS and H_2_.^134,137,138^ The notion of SO_2_ in interstellar ices forming primarily *via* an “energetic” route and OCS forming *via* a “nonenergetic” route is not only in agreement with our present findings, but also with the experimentally demonstrated radiolytic instability of OCS in interstellar ice analogs.^46,87,139^

The production of H_2_SO_4_ hydrates as a result of the irradiation of H_2_O ice on top of sulfur at 70 K was found to have the highest calculated *G*-value (Figure 14 and Table 4), thus highlighting this reaction as an especially productive one. The result of this experiment is in line with those of several previous studies; for instance, early studies by Donaldson and Johnston^140^ and Johnston and Donaldson^141^ investigated the irradiation of colloidal suspensions of elemental sulfur in deaerated H_2_O and found evidence for the solubilization of the sulfur and the subsequent formation of H_2_SO_4_. The mechanism behind this chemistry was later described by Della Guardia and Johnston^123^ (Scheme 1). Perhaps the first work to consider the irradiation of H_2_O:S_8_ mixtures in astrophysical ice analogs was that conducted by Carlson,^142^ who investigated the chemical evolution of S_8_ that was mixed with liquid H_2_O and subsequently frozen prior to irradiation. Their work demonstrated that not only is H_2_SO_4_ efficiently formed by solid-phase radiation chemistry, but that this synthesis is in fact more efficient at 77 K (which is similar to our 70 K irradiation temperature) than at 195 K. Taken together, these results indicate that sulfur allotropes are likely to play a pivotal role in the chemistry of cold outer solar system bodies whose surfaces are characterized by temperatures of *circa* 70 K, such as Jupiter’s moon Europa.^143^

Indeed, on the surface of Europa, allotropic sulfur is thought to be the second-most abundant reservoir of the element, behind H_2_SO_4_ hydrates.^142,144^ Given that the surface of Europa is also dominated by H_2_O ice, our results suggest that the irradiation of this ice on top of deposits of allotropic sulfur could represent a possible route toward the formation of H_2_SO_4_ hydrates and thus could contribute to the so-called radiolytic sulfur cycle taking place on Europa’s surface.^1,45,119,120,125,142,144,145^^,^^146^ It should be noted, however, that the current prevailing hypothesis as to the major source of H_2_SO_4_ hydrates on Europa is the implantation of energetic sulfur ions from the giant Jovian magnetosphere,^125,147,148,149^ which would not only explain the surface abundance of the acid hydrates observed but also their “bulls eye”-like distribution and enhanced presence on the trailing hemisphere.^119,120,150,153,154^ Systematic laboratory work by Ding et al.^125^ has strengthened this hypothesis by demonstrating that the implantation of sulfur ions with kinetic energies of 35–200 keV into H_2_O ice at 80 K results in the production of H_2_SO_4_ with increasing efficiencies at higher ion energies. Sulfur ion implantations into other ices have also been studied both experimentally and theoretically,^151,152,155−162^ and work by Mifsud et al.^151,152^ demonstrated that the implantation of 400 keV sulfur ions into O_2_ ice at 20 K results in the production of SO_2_; this result echoes our present finding with regard to the irradiation of O_2_ ice on top of allotropic sulfur at the same temperature.

In conclusion, our experiments have demonstrated that the irradiation of astrophysical ice analogs on top of refractory sulfur using 1 MeV He^+^ ion beams (used as a mimic of space radiation) results in the formation of various simple inorganic molecules, including SO_2_, CS_2_, OCS, and H_2_SO_4_ hydrates, but notably not H_2_S. The formation of these species from different ices on top of sulfur has been characterized by quantifying their respective *G*-values, and reactions leading to their radiolytic synthesis have been suggested. We emphasize that further work is required to definitively identify the specific reactions leading to the sulfur-bearing products observed in our experiments, including further work on the possible contribution of ion-mediated reactions compared with the neutral–neutral reactions proposed herein. Our results have demonstrated that SO_2_ and CS_2_ are the most commonly observed products among the different ices considered, but that the formation of H_2_SO_4_ hydrates as a result of the irradiation of H_2_O ice on sulfur at 70 K is associated with the highest *G*-value. Finally, we have discussed the astrochemical implications of our work in detail and propose that CS_2_ is a good candidate molecule for future identification in astrophysical ices by observational surveys. Furthermore, we have provided experimental evidence that SO_2_ in interstellar ices could be primarily formed *via* energetic mechanisms while OCS is more likely formed as a result of nonenergetic processes, in agreement with recently published results from experimental and observational studies.
